# Macrophage regulation of the “second brain”: CD163 intestinal macrophages interact with inhibitory interneurons to regulate colonic motility - evidence from the *Cx3cr1-Dtr* rat model

**DOI:** 10.3389/fimmu.2023.1269890

**Published:** 2023-10-05

**Authors:** Jackson L. K. Yip, Soniya Xavier, Gayathri K. Balasuriya, Elisa L. Hill-Yardin, Sarah J. Spencer

**Affiliations:** ^1^ School of Health and Biomedical Sciences, RMIT University, Bundoora, Melbourne, VIC, Australia; ^2^ Department of Physiology and Cell Biology, Kobe University School of Medicine, Kobe, Japan

**Keywords:** gastrointestinal, macrophages, nitric oxide, myenteric plexus, colonic motility

## Abstract

Intestinal macrophages are well-studied for their conventional roles in the immune response against pathogens and protecting the gut from chronic inflammation. However, these macrophages may also have additional functional roles in gastrointestinal motility under typical conditions. This is likely to occur via both direct and indirect influences on gastrointestinal motility through interaction with myenteric neurons that contribute to the gut-brain axis, but this mechanism is yet to be properly characterised. The CX3CR1 chemokine receptor is expressed in the majority of intestinal macrophages, so we used a conditional knockout *Cx3cr1-Dtr* (diphtheria toxin receptor) rat model to transiently ablate these cells. We then utilized *ex vivo* video imaging to evaluate colonic motility. Our previous studies in brain suggested that *Cx3cr1*-expressing cells repopulate by 7 days after depletion in this model, so we performed our experiments at both the 48 hr (macrophage depletion) and 7-day (macrophage repopulation) time points. We also investigated whether inhibitory neuronal input driven by nitric oxide from the enteric nervous system is required for the regulation of colonic motility by intestinal macrophages. Our results demonstrated that CD163-positive resident intestinal macrophages are important in regulating colonic motility in the absence of this major inhibitory neuronal input. In addition, we show that intestinal macrophages are indispensable in maintaining a healthy intestinal structure. Our study provides a novel understanding of the interplay between the enteric nervous system and intestinal macrophages in colonic motility. We highlight intestinal macrophages as a potential therapeutic target for gastrointestinal motility disorders when inhibitory neuronal input is suppressed.

## Graphical Abstract

We used a conditional knockout *Cx3cr1-Dtr* rat model to decipher the role of intestinal macrophages in colonic motility. Transgenic rats injected with diphtheria toxin (DT) showed a significant reduction of ionized calcium-binding adaptor molecule 1 (Iba-1)-positive intestinal macrophages in the myenteric plexus 48 hours post-injection. We demonstrated that in the presence of the nitric oxide synthase inhibitor, Nω-nitro-L-arginine (NOLA), macrophage ablated rats had a greater increase in contraction frequency than controls. We then examined the macrophage population 7 days after DT injection and found that cluster of differentiation 163 (CD163) tissue resident macrophages do not repopulate whereas Iba-1 positive macrophages do. A greater increase in contraction frequency in macrophage-ablated rats in the presence of NOLA indicates that CD163 positive macrophages are crucial in regulating colonic motility in the absence of nitric oxide.

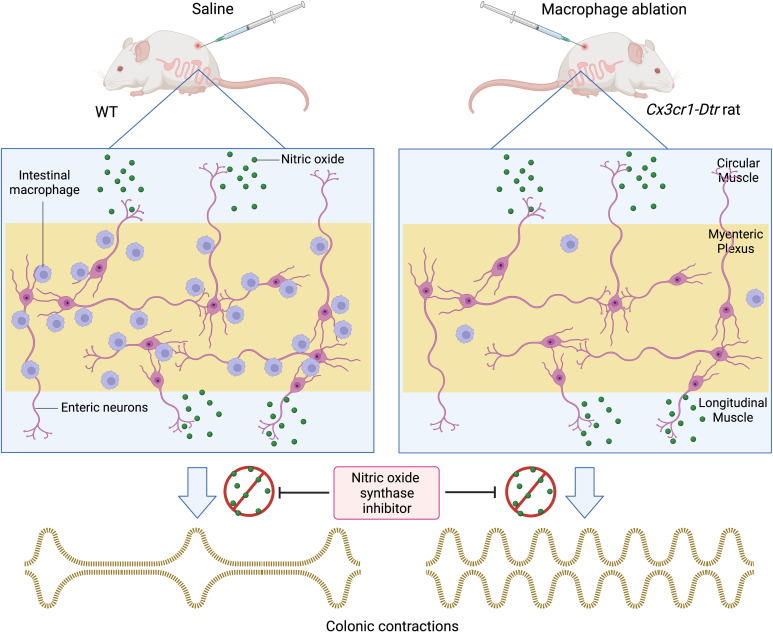

## Highlights

• Intestinal macrophages regulate intestinal motility but the mechanisms by which this occurs are largely unknown.
• We utilized a *Cx3cr1-Dtr* (diphtheria toxin receptor) rat model to transiently deplete macrophages and thus investigate the macrophage contribution to colonic motility in the context of enteric nervous system inhibitory input.
• We show that tissue-resident CD163 intestinal macrophages regulate colonic motility, particularly in the absence of the main inhibitory drive in the gut which occurs via nitric oxide-dependent input.
• These findings allow us to better understand how intestinal macrophages regulate colonic motility and provide insights to support the development of macrophage-specific therapeutic targets for gut motility disorders.

## Introduction

As the most abundant immune cell type of the gastrointestinal tract, intestinal macrophages play a key role in maintaining homeostasis (1), including resistance to invasion by foreign antigens and commensal bacteria. It is accepted that intestinal macrophages generally maintain an anti-inflammatory (M2) profile to prevent chronic inflammation and promote tissue repair (2, 3). They secrete the anti-inflammatory cytokine interleukin (IL)-10, which is constitutively expressed in the gut in the healthy individual (3). During inflammation, intestinal macrophages differentiate into pro-inflammatory macrophages (M1) and secrete pro-inflammatory cytokines, including IL-1β, IL-6, and tumour necrosis factor (TNF)-α (1). The release of pro-inflammatory cytokines helps combat pathogens by further recruitment of inflammatory cells or by stimulating production of inflammatory response proteins such as serum amyloid A and C-reactive protein (4, 5).

In addition to an immunological role, intestinal macrophages regulate intestinal motility via interaction with the enteric nervous system (ENS), specifically with myenteric neurons. Muller and others have demonstrated that colony stimulating factor 1 (CSF-1)/bone morphogenetic protein 2 (BMP-2) bi-directional signaling between enteric neurons and intestinal macrophages is crucial in maintaining intestinal peristalsis in healthy mice (6). However, the mechanisms by which this neuroimmune crosstalk affects intestinal muscle contractions, and thereby digestion, are still poorly understood (3). Here we hypothesized that gastrointestinal motility would be broadly maintained in the absence of intestinal macrophages but that motility responses to ENS input would lack the coordinated contractile patterns seen typically in rodents, highlighting the interplay between the gut-brain axis and its immune component.

Different myenteric neuron populations, as identified by their neurochemical coding, play different roles in regulating intestinal motility (7). The major inhibitory myenteric neurons express neuronal nitric oxide synthase (nNOS) (8). nNOS neurons stimulate relaxation of the smooth muscle (9). A recent study has suggested that colonic migrating motor complexes (CMMCs), responsible for initiating colonic contractions, originate from the blockade of the inhibitory nitrergic cyclic guanosine monophosphate (cGMP)-dependent pathway (10). The nitrergic pathway is therefore likely to play a principal role in regulating colonic motility (10). As such, loss of nNOS has been implicated in several gastrointestinal disorders, such as oesophageal achalasia, gastroparesis and Hirschsprung’s disease (11–13). In mice, transplanting healthy enteric neural stem cells into nNOS-deficient mice can rescue impaired colonic motility (7).

Although colonic motility is regulated by the neurons of the myenteric plexus, additional factors contribute to the detailed contraction profile (14). Apart from input by myenteric neurons, pacemaker interstitial cells of Cajal also generate myogenic rhythmicity (14). Depending on the distance that motor complexes travel, the resultant neurogenic and myogenic contractions in the proximal colon can be characterized into different patterns (15). Therefore, in this study we focused on defining differences in contraction patterns occurring in the rat proximal to mid colon following the ablation of intestinal macrophages.

One of the main identifiers of intestinal macrophages is the CX3C chemokine receptor 1 (Cx3cr1). *Cx3cr1* expression is low in circulating monocytes but increases as monocytes differentiate into resident intestinal macrophages (3, 16). Therefore, a transgenic model targeting *Cx3cr1*-expressing cells allows us to directly investigate the role of intestinal macrophages in the gut. In previous work, we used a conditional diphtheria toxin receptor (Dtr) knock-in *Cx3cr1-Dtr* rat model to target Cx3cr1-containing cells and study their roles in satiety control, circadian rhythms, neuroimmune responses and cognitive function (17–21). These studies suggest that the effects of ablating Cx3cr1-cells (i.e., microglia, monocytes, macrophages) are not due to sickness, withdrawal, anxiety, or nausea (17). Here, we utilized this transgenic rat model to investigate the role of intestinal macrophages in gastrointestinal motility and their interactions with the ENS. We measured colonic motility patterns at the mid-point of the proximal colon, since previous studies demonstrated that only a subset of contractions generated from the beginning of the rat proximal colon are propagated into mid-colon and beyond (22–25). We found that the loss of intestinal macrophages in this model led to shortening of the small intestine and colon. Furthermore, intestinal macrophage depletion increased motility in the proximal colon only when nNOS was inhibited. The difference in motility was not caused by changes in the number of nNOS neurons in the myenteric plexus. Notably, spontaneous repopulation of ionized calcium binding adaptor molecule 1 (Iba-1)-positive but not cluster of differentiation 163 (CD163)-positive intestinal macrophages ensued after 7 days, and this was sufficient to rescue some aspects of the phenotype, including intestine length. However, we observed increased motility upon nNOS inhibition that persisted even after Iba-1-positive macrophages had repopulated the tissue. Our findings indicate that CD163-positive macrophages are crucial in regulating gut motility when the major inhibitory neural input is blocked.

## Methods

### Animals

All experiments were conducted in accordance with the Australian Code of Practice for the Care and Use of Animals for Scientific Purposes, with approval from the RMIT University Animal Ethics Committee (AEC #1920). The chemokine receptor Cx3cr1 is exclusively expressed in microglia and monocytes (26). To specifically ablate Cx3cr1-expressing cells, we generated a *Cx3cr1-Dtr* knock-in rat model on a Wistar Han background using CRISPR/Cas9 technology, as previously described (17).

In the present experiments, we used female rats aged between 13 and 17 weeks. Initial analyses suggested females perform similarly to males in terms of microglial and weight responses to the DT (17) and so we selected one sex only to first establish mechanistic insight into how macrophages affect gut motility before proceeding to sex-comparison studies. The rats were kept under standard laboratory housing conditions, with a 12 hr light cycle (7 am to 7 pm), an ambient temperature of 22 °C, with humidity between 40 and 60%, and free access to water and standard rat chow except where stated. We administered DT as two separate injections, 8 hr apart, of 25 ng/g DT in sterile saline, subcutaneously (s.c.), according to our previous studies (17, 27). Our previous work has shown that depletion of microglia and monocytes is maximized at 48 hr, and that spontaneous repopulation is in progress around 7 days after depletion (17). Thus, basal and post-DT tissue collection was performed 48 hr or 7 days after the first injection, after the rats were euthanized with overdose of ketamine and xylazine, 20 mg/mL ketamine (Cenvet Australia, Lynbrook, VIC, Australia), 5 mg/mL xylazine (Cenvet). All experiments were completed between 9 am and 1 pm to limit potential effects of circadian rhythms on any parameters measured.

### Colon collection and wholemount tissue preparation for immunofluorescence

The proximal colon (the first 3-5 cm of colon measured from the caecum and visualized by colonic striation patterns) from each animal was opened, stretched, pinned with the mucosa facing upwards, and submerged in 0.1 M phosphate buffered saline (PBS) on a Petri dish lined with Sylgard (Sylgard Silicone Elastomer, Krayden Inc., Denver, CO, USA). To obtain a longitudinal muscle-myenteric plexus (LMMP) preparation, the mucosa, submucosal plexus and circular muscle were peeled away from the remaining colonic tissue under a dissecting microscope. A small area of tissue containing the LMMP was transferred to a Petri dish (35 mm), submerged in 0.1 M PBS, and stored at 4°C before assessment of neuronal populations by immunofluorescence.

### Wholemount immunofluorescence for neuronal populations and identification of intestinal macrophages

We have previously described the myenteric plexus wholemount immunofluorescence for mouse tissues (28). Here, immunofluorescence was performed on wholemount rat colonic tissue samples to assess for potential differences in neuron numbers and intestinal macrophage populations between saline- and DT-treated *Cx3cr1-Dtr* rats. Wholemount LMMP samples were incubated at room temperature (RT) for 30 min in 0.01% Triton X-100 (Sigma Aldrich, St Louis, MO, USA) with 10% CAS-block™ (Invitrogen Australia, Mt Waverley, VIC, Australia) to reduce non-specific binding of antibodies. Then, tissues were incubated with three primary antisera for neuronal populations: human anti-Hu (1:5,000, a pan-neuronal marker; a gift from Dr. V. Lennon, Mayo Clinic, Rochester, MN, USA), sheep anti-nNOS (1:400; Millipore, RRID: AB_90743) and rabbit anti-Iba-1 (1:400; Wako Chemicals USA Inc., Richmond, VA, USA, RRID: AB_839504) and stored at 4°C overnight in a sealed container. For assessing macrophage populations, tissues were incubated with two primary antisera: rabbit anti-Iba-1 (FUJIFILM Wako Shibayagi, RRID: AB_839504; 1:400) and mouse anti-CD163 (Bio-Rad Laboratories, RRID: AB_2074558; 1:100). After incubation, colonic tissues were washed with 0.1 M PBS (three washes of 10 min each). Secondary antisera corresponding to the host of the primary antibody were applied to the samples and left for 2.5 hr at RT on a shaker incubator (donkey anti-sheep Alexa 488 (Thermo Fisher Scientific, RRID: AB_2534082); 1:400, donkey anti-human Alexa 594 (Jackson ImmunoResearch Laboratories, Inc., RRID: AB_2340572); 1:750, donkey anti-rabbit Alexa 647 (Jackson, RRID: AB_2340572); 1:400 and donkey anti-mouse Alexa 488 (Abcam, RRID: AB_2732856)). Colonic tissues were mounted using fluorescence mounting medium (DAKO Australia Private Ltd; Botany, NSW, Australia). Tissue samples were imaged using a confocal microscope (Nikon Confocal Microscope: A1; Version 4.10). A Z-series of images of myenteric plexus sections (6.5 μm/step with total tissue thickness approximately 60 μm) was captured for each animal and saved in the ND2 file format.

### Analysis of nNOS neuron populations in the myenteric plexus

Images of colonic tissue containing the myenteric plexus were analysed using ImageJ (1.52a, NIH, Bethesda, MD, USA). Five intact myenteric ganglia were randomly selected from each wholemount colonic tissue sample (approximately 1 cm^2^) for each animal. We then counted the number of Hu- and nNOS-labelled cells from each ganglion. The nNOS neuronal population was estimated as the percentage of nNOS cells in a ganglion co-labelled with Hu.

### Intestinal macrophage density and morphology

Z-series images of wholemount tissue were analysed using the Imaris software volume function to assess the cell density and morphology of intestinal macrophages (Imaris 64X 9.1.0; Bitplane AG, UK). Three proximal colon areas of 0.25 mm^2^ per tissue per animal were selected as regions of interest (ROI). The presence of macrophages in the muscle layer was established by visualising the z-position of Iba-1-positive cells (macrophages) relative to that of Hu-positive cells (neurons). Macrophages with a z-position outside the location of the neurons were considered to be situated in the muscle layer within the LMMP preparation. Sphericity and cell density data were also recorded and analysed using GraphPad Prism software (Boston, MA, USA; version 9.0.1).

### RT-PCR

Proximal colons were snap-frozen and RNA extracted using QIAzol reagents and RNeasy Mini Kits (Qiagen, Valencia, CA, USA). RNA was then transcribed to cDNA using Quantitect Reverse Transcription kits (Qiagen) and analyzed by qRT-PCR with a QuantStudio 7 Flex instrument (Applied Biosystems, Mulgrave, Vic, Australia) using Taqman Gene Expression Assays (Applied Biosystems). We compared the relative quantitative measure of *Cx3cr1* expression (NCBI reference sequence: NM_133534.1, Taqman assay ID: Rn02134446_s1) with the housekeeping gene *Gapdh* (NCBI reference sequence: NM_017008.3, Taqman assay ID: 4352338E) as an endogenous control. We analysed mRNA expression using 2^−ΔΔC(t)^, where C(t) is the threshold cycle at which fluorescence is first detected significantly above background.

### *Ex vivo* video imaging of colonic motility

The setup for rat colon has been described in our previous publication (25). Briefly, the proximal to mid colon (5-7 cm measured from the caecum end) was dissected from each animal. Each colon preparation was placed into a beaker containing Krebs solution (118 mM NaCl, 4.6 mM KCl, 2.5 mM CaCl_2_, 1.2 mM MgSO_4_, 1 mM NaH_2_PO_4_, 25 mM NaHCO_3_, 11 mM D-glucose in mM; bubbled at RT with carbogen gas: 95% O_2_ and 5% CO_2_) at 4°C. The colon preparation was then placed into an organ bath chamber, which was connected to an in-flow reservoir containing Krebs solution via inlet tubes and was continuously superfused with Krebs solution bubbled with carbogen and maintained between 33-35°C. The oral end of the colon preparation was cannulated to the inlet tube and secured using standard cotton sewing thread. The faecal content was removed by applying gentle positive pressure from the inflow reservoir. The anal end of colon was then cannulated to the outlet tube. An intraluminal pressure was created by using a rubber stopper with a glass tube (5 mm inside diameter) inserted through its centre to seal onto the inflow reservoir. Intraluminal pressure was calculated by measuring the vertical distance from the tissue to the meniscus of Krebs solution within the glass tube of inflow reservoir and maintained at constant level throughout the experiment (i.e., the meniscus was 5.5-6.5 cm above the height of the colon segment). Colonic motility was recorded using a Logitech camera (QuickCam Pro 4000; I‐Tech, Ultimo, NSW, Australia) mounted directly above the organ bath at a standard distance of 10 cm. Each colon was given 30 min to equilibrate before we recorded four 15 min videos of spontaneous contractile activity under control conditions. Subsequently, 100 μM Nω-nitro-L-arginine (NOLA) was added to the inflow reservoir, to inhibit nitric oxide, and contractile activity was recorded for another four x 15 min. After NOLA application, a final four x 15 min videos were recorded, considered as the washout period. These final recordings enabled us to assess the restoration of the inhibitory stimulus and to ensure the tissue remained viable for the duration of the experiment.

### Pair-feeding motility

Transient ablation of macrophages in the *Cx3cr1-Dtr* causes anorexia-induced weight loss while the macrophages remain depleted (17). Therefore, to verify that any changes in intestinal motility were due to the absence of intestinal macrophages and not to any anorexia or weight loss that accompanies it, we performed a pair-feeding experiment (17). We fed a cohort of macrophage-intact rats the mean voluntary consumption of the DT-treated *Cx3cr1-Dtr* rats to induce a similar weight loss to that associated with the macrophage ablation. Rats were then anaesthetized for tissue collection and assessment of colonic motility as described above.

### Statistical analysis

Statistical analyses were performed using GraphPad Prism software (GraphPad; version 9.0.1). We assessed nNOS neuronal populations, cell density and sphericity of intestinal macrophages, RT-PCR, percentage changes of resting gut diameter, contraction magnitude and contraction frequency before and after NOLA treatment using Student’s unpaired t-tests. We assumed statistical significance when *p* < 0.05. A repeated measures two-way analysis of variance (ANOVA) was used to compare the resting gut diameter, contraction magnitude and contraction frequency of macrophage-intact and *Cx3cr1-Dtr* rat colon under control conditions and with NOLA treatment. Tukey *post hoc* tests were used to identify where significant differences occurred in the case of a significant interaction. Data are presented as mean with maximum and minimum. Sample sizes are included in the individual results sections.

## Results

### Macrophage ablation reduces Iba-1-positive cell density in the myenteric plexus

**Figure 1:**
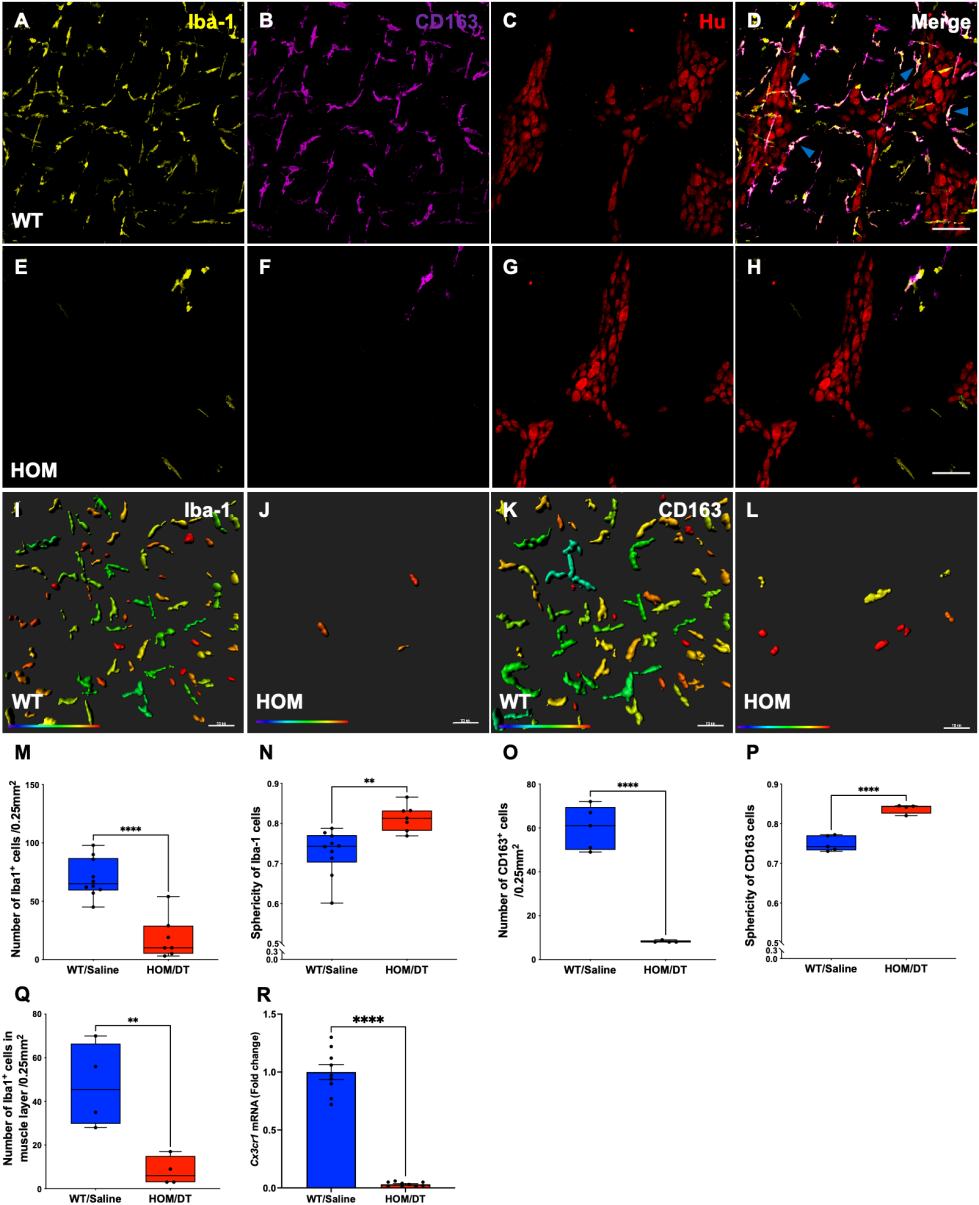
*Cx3cr1*-cell ablation significantly reduced the density and increased the sphericity of Iba-1 cells and CD163 cells in the myenteric plexus of the proximal colon. **(A)** Macrophage-intact proximal colon myenteric plexus ganglia immunolabelled with ionized calcium binding adaptor molecule 1 (Iba-1), **(B)** cluster of differentiation 163 (CD163) and **(C)** Hu. **(D)** Iba-1, CD163 and Hu merged. **(E)** *Cx3cr1-Dtr* rat proximal colon 48 hr after diphtheria toxin (DT) immunolabelled with Iba-1, **(F)** CD163 and **(G)** Hu. **(H)** Iba-1, CD163 and Hu merged. **(I)** Imaris colour-gradient of sphericity of Iba-1 cells in macrophage-intact proximal colon and **(J)** *Cx3cr1-Dtr* rat proximal colon. **(K)** Imaris colour-gradient of sphericity of CD163 cells in macrophage-intact proximal colon and **(L)** *Cx3cr1-Dtr* rat proximal colon. **(M)** Numbers of Iba-1-expressing cells per 0.25 mm^2^. **(N)** Cx3cr1-cell ablation leads to a significant increase in the sphericity of Iba-1-positive cells in the myenteric plexus of *Cx3cr1-Dtr* rats given DT compared to WT. **(O)** Numbers of CD163-expressing cells per 0.25 mm^2^. **(P)** Cx3cr1-cell ablation leads to a significant increase in the sphericity of CD163-positive cells in the myenteric plexus of *Cx3cr1-Dtr* rats given DT compared to those not given DT. **(Q)** Number of Iba-1-positive cells in the muscle layer per 0.25mm^2^. **(R)** *Cx3cr1* gene expression in proximal colon of *Cx3cr1-Dtr* rats given DT compared to those not given DT. Data are mean with maximum and minimum. ** *p* ≤ 0.01, **** *p* ≤ 0.0001, Scale bar = 100 μm for confocal images, 70 μm for Imaris colour-gradient images. Sphericity colour gradient scale = 0.2-0.9; red indicates more spherical, blue indicates more elongated.

To verify that intestinal macrophages are depleted upon DT injection in *Cx3cr1-Dtr* rats, we assessed numbers of Iba-1-positive cells in the myenteric plexus. As expected, at 48 hr after DT injection there was significant loss of intestinal macrophages in the myenteric plexus (t_(15)_ = 6.09, *p* < 0.0001, n = 7-10 animals per group; **Figures 1A, E, M**). Resident intestinal macrophages are particularly important in the bidirectional communication between the nervous and immune systems (29, 30), so we also assessed the CD163-positive (resident) subpopulation of macrophages. Notably, most intestinal macrophages surrounding ganglia expressed CD163 (**Figures 1C, D**, blue arrows). Similar to Iba-1-expressing cells, CD163-positive cells were ablated upon DT injection (t_(7)_ = 10.25, *p* < 0.0001, n = 4-5 animals per group; **Figures 1B, F–H, O**). Iba-1-positive cells in the smooth muscle layers were also significantly reduced after DT (t_(6)_ = 3.85, *p* = 0.0085, n = 4 per group; **Figure 1Q**). Additionally, *Cx3cr1* mRNA was drastically reduced after DT injection to be almost undetectable (t_(16)_ = 14.9, *p* < 0.0001, n = 9 per group; **Figure 1R**), verifying the efficacy of our model in depleting its target cells. Colocalization analysis indicated that about 70% of Iba-1 positive macrophages in the rat colon also expressed CD163.

As anticipated from our previous work in the brain (18), the morphology of the macrophages remaining after depletion differed from macrophages in intact rats, with both Iba-1-positive cells and CD163-positive cells being significantly more spherical in the *Cx3cr1-Dtr* rats than in those not given DT (Iba-1-positive cells: t_(15)_ = 3.60, *p* = 0.0026, n = 7-10 animals per group, **Figures 1I, J, N**; CD163-positive cells: t_(7)_ = 7.89, *p* < 0.0001, n = 7-10 animals per group, **Figures 1K, L, P**).

### Macrophage ablation decreases body weight and shortens small intestine and colon

**Figure 2:**
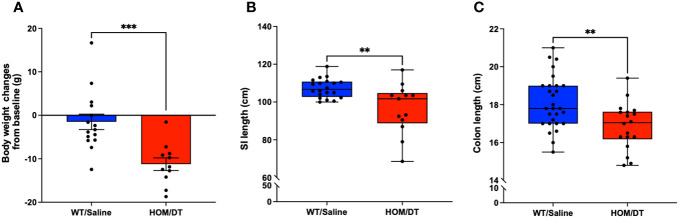
Macrophage-ablated *Cx3cr1-Dtr* rats have reduced body weight, small intestinal and colon length. Depletion of microglia and monocytes leads to significantly reduced **(A)** body weight, **(B)** small intestine length and **(C)** colon length in *Cx3cr1-Dtr* rats 48 hr after diphtheria toxin (DT) injection, relative to macrophage-intact rats. Data are mean with maximum and minimum. ** *p* ≤ 0.01 and *** *p* ≤ 0.001.

After verifying the conditional knockout of intestinal macrophages in *Cx3cr1-Dtr* rats, we investigated if the loss of intestinal macrophages affected the overall anatomy of the gastrointestinal tract. Consistent with previous findings from our group (17), the body weights of *Cx3cr1-Dtr* rats were significantly reduced at 48 hr after DT injection (t_(24)_ = 4.03, *p* = 0.0005, n = 11-15 animals per group; **Figure 2A**). We also found that both small intestine (t_(31)_ = 3.32, *p* = 0.002, n = 13-20 animals per group; **Figure 2B**) and colon length (t_(44)_ = 3.09, *p* = 0.003, n = 18-28 animals per group; **Figure 2C**) were shortened by intestinal macrophage ablation.

### Effects of macrophage depletion on colonic motility

**Figure 3:**
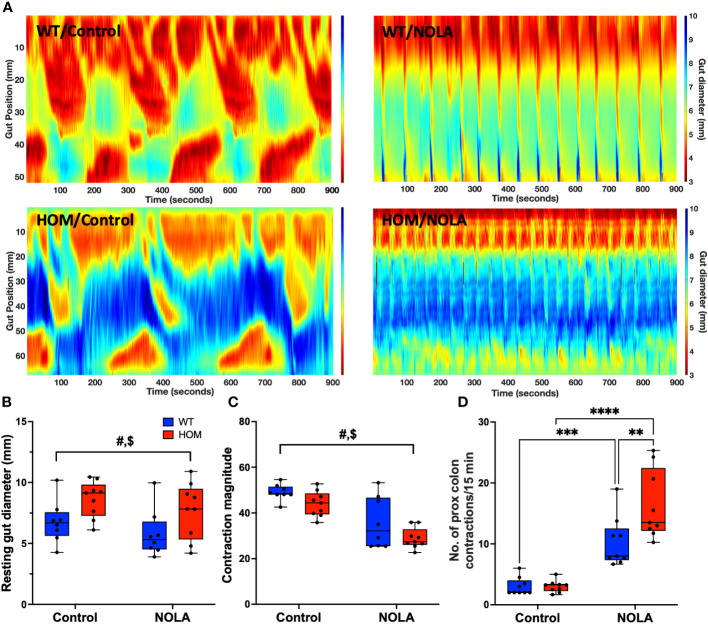
Intestinal macrophages regulate, but are not necessary for, colonic motility. **(A)** Representative spatiotemporal heatmaps of macrophage-intact (WT) and *Cx3Cr1-Dtr* rats under control conditions and with Nω-nitro-L-arginine (NOLA) treatment. **(B)** Resting gut diameter of control and macrophage-ablated rats. **(C)** Contraction magnitude of control and *Cx3Cr1-Dtr* rats. **(D)** Contraction frequency per 15 min in the proximal colon of control and macrophage-ablated rats. # two-way ANOVA with main effect of genotype, $ main effect of NOLA treatment, ** Tukey *post-hoc* test *p* < 0.01, *** *p* < 0.001, **** *p* < 0.0001.

Next, we examined if the loss of intestinal macrophages in the myenteric plexus had any impact on colonic motility. We measured the resting gut diameter, contraction magnitude and frequency of contraction in the proximal colon as described previously (25). Our results demonstrated that macrophage-ablated rats have a wider resting colon diameter than macrophage-intact rats under control conditions (i.e., without NOLA; main effect of genotype: F_(1,15)_ = 7.16, *p* = 0.0017, n = 8-9 animals per group, **Figures 3A, B**). The loss of intestinal macrophages also led to a decrease in contraction magnitude (main effect of genotype: F_(1,15)_ = 45.0, *p* < 0.0001, n = 8-9 animals per group, **Figure 3C**), suggesting the importance of intestinal macrophages in colonic motility under control conditions. On the other hand, inhibiting nNOS (via NOLA treatment) stimulated smooth muscle contraction and thus reduced resting gut diameter (main effect of NOLA: F_(1,15)_ = 4.85, *p* = 0.044, n = 8-9 animals per group, **Figures 3A, B**), but caused a decrease in contraction magnitude (main effect of NOLA: F_(1,15)_ = 5.69, *p* = 0.031, n = 8-9 animals per group, **Figure 3C**). As expected, inhibiting nNOS increased the frequency of contractions in the proximal rat colon. However, we observed a significantly higher increase in contraction frequency in macrophage-ablated rats (interaction effect: F_(1,16)_ = 7.84, *p* = 0.013, n = 8-9 animals per group, NOLA effect on macrophage-intact rat colon: p = 0.0008, NOLA effect on *Cx3cr1-Dtr* rat colon: p < 0.0001, **Figure 3D**). The increase in contraction frequency upon inhibition of nNOS indicates that intestinal macrophages can regulate colonic motility without major inhibitory neuronal input.

### Effect of pair-feeding on colonic motility

**Figure 4:**
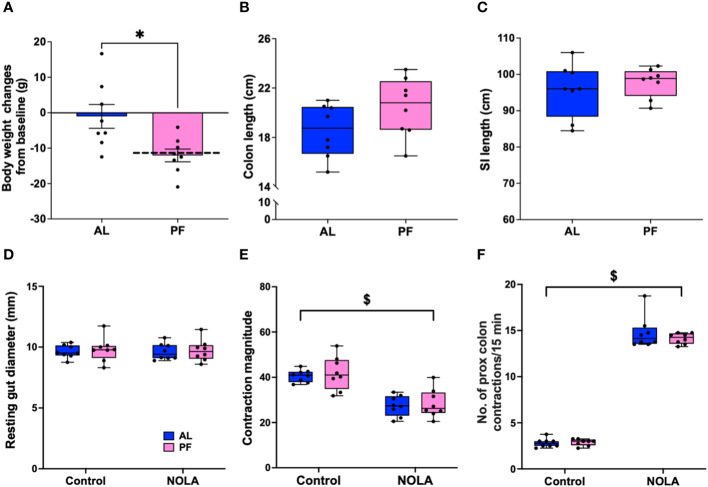
Food restriction does not affect colonic motility. **(A)** Body weight changes between *ad libitum*-fed rats (AL) and macrophage-intact (WT) rats pair-fed (PF) to that consumed by the diphtheria toxin (DT)-injected *Cx3cr1-Dtr* rats (PF), dotted line indicated the body weight changes of *Cx3cr1-Dtr* rats 48 hrs after DT injection. **(B)** Colon length of AL and PF rats. **(C)** Small intestine length of AL and PF rats. **(D)** Resting gut diameter of AL and PF rats under control conditions and with NOLA treatment. **(E)** Contraction magnitude of AL and PF rats under control conditions and with NOLA treatment. **(F)** Contraction frequency in 15 min of AL and PF rats under control conditions and with NOLA treatment. $ two-way ANOVA with main effect of NOLA treatment. * p < 0.05.

It has been reported that acute fasting or restricted energy intake may have a direct or indirect effect on gastrointestinal motility through satiety hormones (31–33). In our *Cx3cr1-Dtr* rats, we have consistently reported a decrease in body weight (**Figure 2A**) as well as food intake (17) upon DT injection. Therefore, we performed a pair-feeding experiment to verify if the changes we saw in intestinal structure and colonic motility were macrophage-related or were instead due to a decrease in food intake. As expected, the pair-fed macrophage-intact rats had a significant decrease in body weight when compared to controls (t_(14)_ = 2.90, *p* = 0.01, n = 8 animals per group, **Figure 4A**). Compared with previous weight changes from *Cx3cr1-Dtr* rats 48 after DT injection (dotted line, **Figure 4A**), indicating weight loss after DT injection was largely due to reduced food intake. Interestingly, there were no differences in the length of the small intestine or colon between pair-fed rats and *ad libitum*-fed, macrophage-intact, controls (**Figures 4B, C**). In terms of resting gut diameter, contraction magnitude and contraction frequency, we also did not observe any significant differences between pair-fed and *ad libitum*-fed, macrophage-intact, control rats (**Figures 4D–F**), together indicating a macrophage-specific effect on colonic motility rather than one related to food intake or changes in digestion.

### Macrophage ablation does not affect neuron numbers in the myenteric plexus

**Figure 5:**
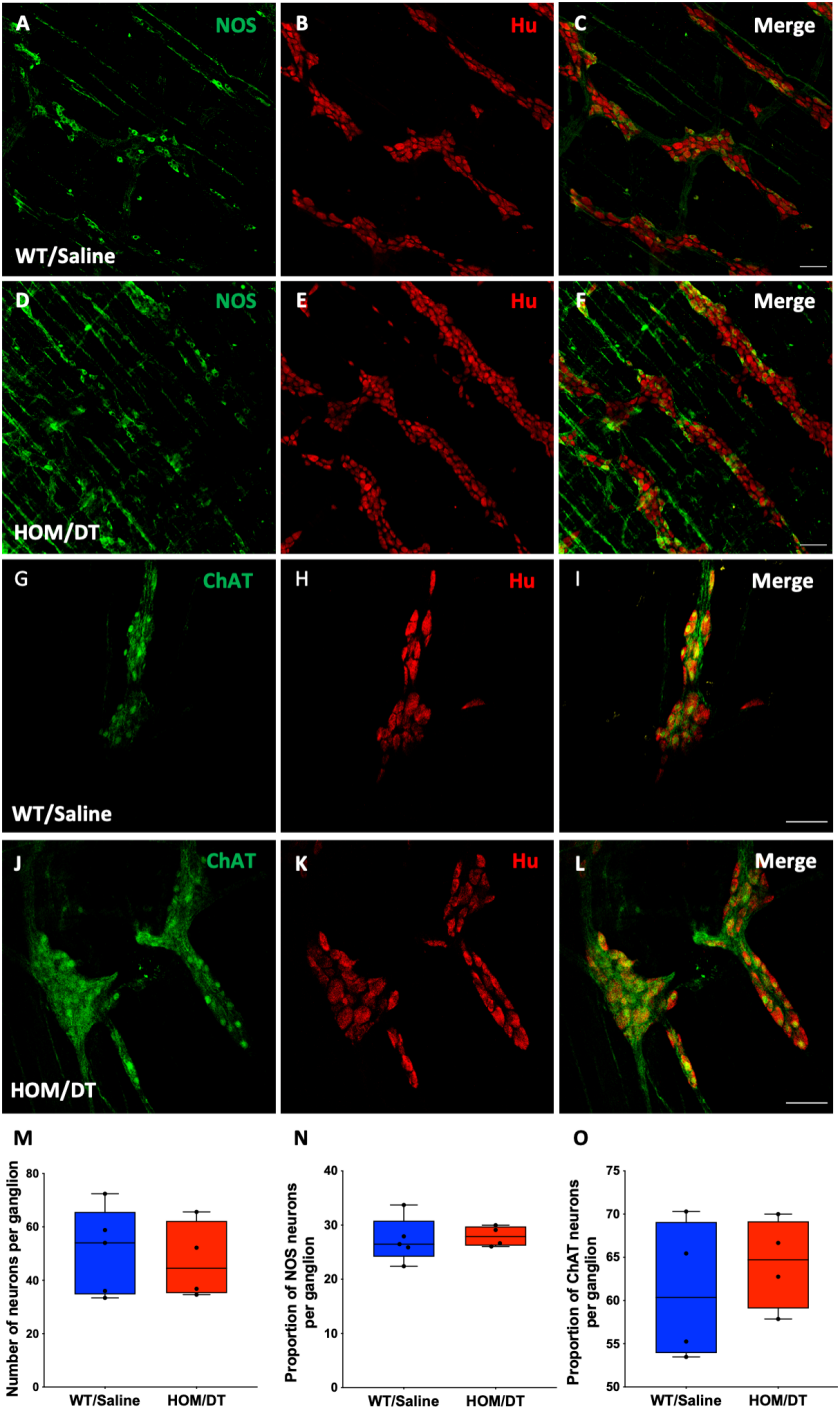
Cx3cr1 ablation does not affect the number of neurons in the myenteric plexus of the rat proximal colon. **(A–C)** control proximal colon myenteric plexus ganglia immunolabelled with **(A)** neuronal nitric oxide synthase (nNOS), **(B)** Hu and **(C)** nNOS and Hu merged. **(D–F)** *Cx3cr1-Dtr* rat proximal colon myenteric plexus ganglia immunolabelled with **(D)** nNOS, **(E)** the pan-neuronal marker, Hu, **(F)** nNOS and Hu merged. **(G–I)** control proximal colon myenteric plexus ganglia immunolabelled with **(G)** choline acetyltransferase (ChAT), **(H)** Hu and **(I)** ChAT and Hu merged. **(J–L)** *Cx3cr1-Dtr* rat proximal colon myenteric plexus ganglia immunolabelled with **(J)** ChAT, **(K)** Hu, **(L)** ChAT and Hu merged. **(M)** Macrophage ablation does not affect the number of neurons (Hu-labelled cells) per ganglion, **(N)** the number of nNOS or **(O)** ChAT neurons per ganglion. Data are displayed as mean with maximum and minimum values. Scale bars = 100 μm.

Neurons in the myenteric plexus are mainly responsible for regulating colonic motility (3, 15). Based on our findings that the loss of intestinal macrophages led to an increase in colonic motility in the absence of the major inhibitory neuronal input (i.e. with NOLA), we assessed whether the size or proportion of the population of nNOS-expressing neurons in the myenteric plexus was changed in response to the loss of macrophages. The number of myenteric neurons per ganglion remained unchanged upon macrophage ablation (**Figures 5B, E, M**), consistent with the findings from De Schepper et al., who showed, in an embryonic macrophage-depletion model that apoptosis of neurons caused by the loss of intestinal macrophages does not take place in mice until day 7 (30). There was also no significant change in the number of neurons expressing nNOS per ganglion in the myenteric plexus (**Figures 5A, C, F, N**). Proportions of acetylcholinergic (ChAT)-expressing neurons within the myenteric plexus were also unaffected (**Figures 5G–L, O**).

### Iba-1- but not CD163-positive macrophages repopulate 7 days after DT injection

**Figure 6:**
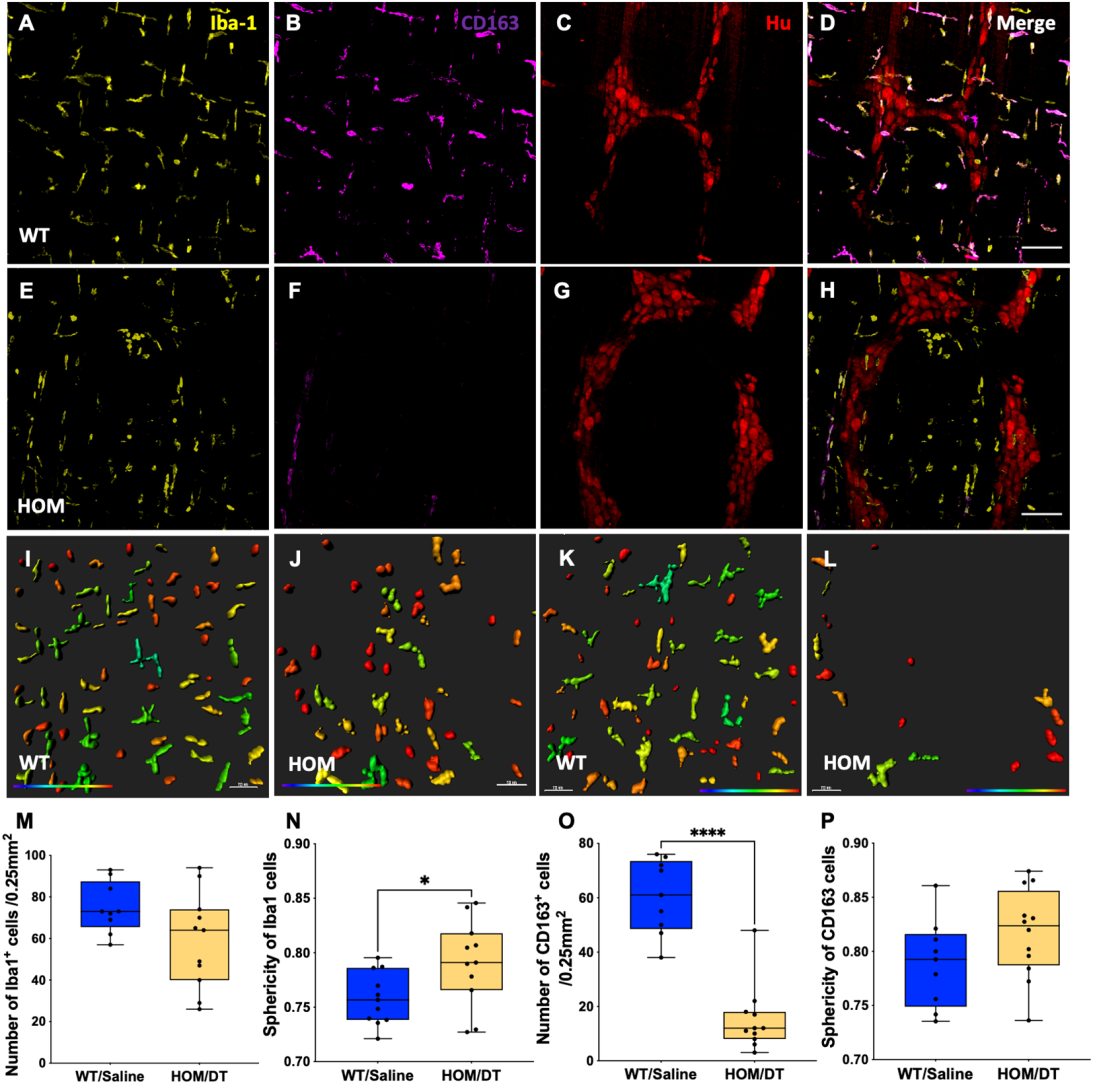
Iba-1- but not CD163-positive macrophages repopulate 7 days after DT injection. Proximal colon myenteric plexus was immunolabelled with **(A, E)** Ionized calcium-binding adaptor molecule 1 (Iba-1), **(B, F)** cluster of differentiation 163 (CD163) and **(C, G)** Hu. **(D, H)** Iba-1, CD163 and Hu (merged). **(A–D)** Controls. **(E–H)** Diphtheria toxin (DT)-injected *Cx3cr1-Dtr*. **(I–L)** Imaris colour-gradient of the sphericity of **(I, J)** Iba-1 and **(K, L)** CD163 cells in the **(I, K)** control and **(J, L)** DT-injected *Cx3cr1-Dtr* rat proximal colon. **(M)** Macrophage ablation did not affect the number of Iba-1-positive cells per 0.25 mm^2^. **(N)** Sphericity of Iba-1 positive cells remained higher in *Cx3cr1-Dtr* rats than in controls 7 days after macrophage ablation. **(O)** CD163-positive cell numbers remained reduced at 7 days after macrophage ablation in *Cx3cr1-Dtr* rats compared with controls and **(P)** sphericity was unchanged. Data are mean with maximum and minimum, * *p* ≤ 0.05, *** *p* ≤ 0.001. Scale bars = 100 μm. 70 μm for Imaris colour-gradient images. Cellular sphericity colour gradient scale = 0.2-0.9; red indicates more spherical, blue indicates a more elongated cellular morphology.

We next investigated whether intestinal macrophages repopulate after depletion, and if this could rescue some of the effects on colonic motility. We previously reported that microglia are repopulating the brain by 7 days after DT injection in the *Cx3cr1-Dtr* model (17). In accordance with this, we observed that Iba-1 expressing cells had repopulated the proximal colon at this time (**Figures 6A, C–E, G, H, M**). Although similar numbers of Iba-1-expressing cells were present in the colon 7 days following DT injection, the morphology of these cells remained more rounded in *Cx3cr1-Dtr* rats, similar to that of 48 hr after ablation (t_(20)_ = 2.35, *p* = 0.029, n = 11 animals per group; **Figures 6I, J, N**). We also used CD163 as a marker to assess tissue-resident macrophage repopulation. Interestingly, CD163-positive macrophages remained depleted at this 7-day time point (t_(8)_ = 6.94, *p* = 0.0001, n = 11 animals per group; **Figures 6B–D, F–H, O**) without a change in sphericity (**Figures 6K, L, P**).

### Macrophage repopulation recovers colon and small intestine length

**Figure 7:**
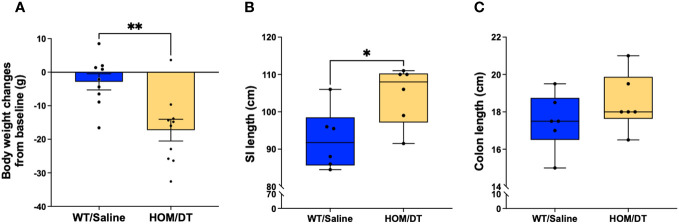
Macrophage repopulation leads to increased small intestine length and recovered colon length at 7 days after macrophage loss. **(A)** DT-injected *Cx3cr1-Dtr* rats maintained reduced body weight 7 days after macrophage ablation. **(B)** *Cx3cr1-Dtr* rats had significantly longer small intestines than controls 7 days after macrophage ablation. **(C)** There was no significant difference in colon length between controls and *Cx3cr1-Dtr* rats 7 days after macrophage ablation. Data are depicted as mean with maximum and minimum values. *p* ≤ 0.05. * *p* ≤ 0.05 and ** *p* ≤ 0.01.

After confirming that Iba-1-positive macrophages repopulate 7 days after depletion as expected, we next investigated if the gastrointestinal anatomical phenotypes we observed were also reversed at this timepoint. There remained a persistent reduction in body weight in macrophage-ablated rats (F_(5,5)_ = 1.02, *p* = 0.005, n = 9-10 animals per group; **Figure 7A**). However, the difference in colon length was no longer evident (F_(5,5)_ = 1.04, *p* = 0.28, n = 6 animals per group; **Figure 7C**) and the small intestine length was significantly longer in the *Cx3cr1-Dtr* rats 7 days after DT injection than in controls (F_(5,5)_ = 1.08, *p* = 0.027, n = 6 animals per group; **Figure 7B**). Thus, the repopulation of Iba-1-positive intestinal macrophages was sufficient to rescue the shortened small intestine and colon associated with macrophage loss.

### Macrophage repopulation effects on colonic motility

**Figure 8:**
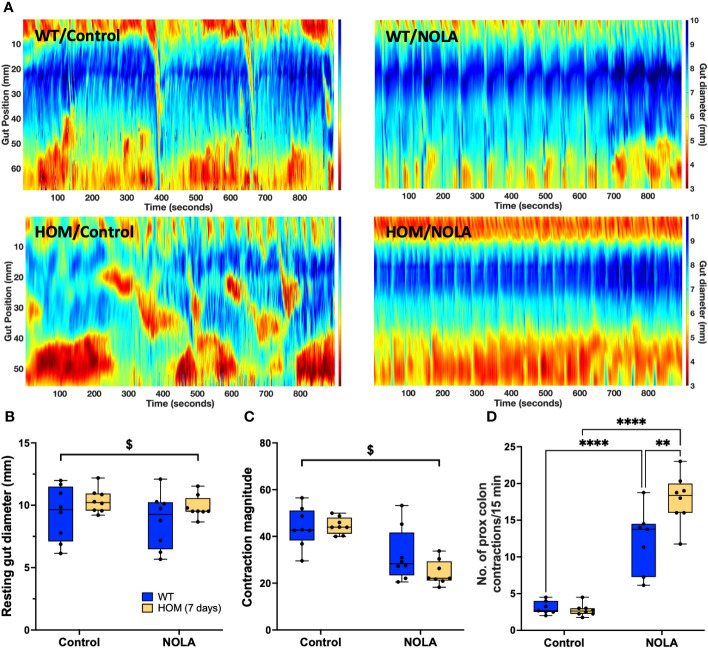
Iba-1-positive macrophage repopulation does not rescue motility dysregulation in the absence of inhibitory neuronal input. **(A)** Representative spatiotemporal heatmaps of control (WT) and *Cx3Cr1-Dtr* rats under control conditions and with Nω-nitro-L-arginine (NOLA) treatment. **(B)** Resting gut diameter of control and macrophage-repopulating rats. **(C)** Contraction magnitude of control and *Cx3Cr1-Dtr* rats. **(D)** Contraction frequency per 15 min in the proximal colon of control and macrophage-repopulating rats. # two-way ANOVA with main effect of genotype, $ main effect of NOLA treatment, ** Tukey *post-hoc* test *p* < 0.01, **** *p* < 0.0001.

As we observed that the shortened colon and small intestinal phenotypes were rescued upon the repopulation of Iba-1-positive-only intestinal macrophages, we examined if the exacerbation of the NOLA-induced changes in resting gut diameter and contraction frequency caused by macrophage ablation were similarly restored. There was no significant difference, however, in resting gut diameter and contraction magnitude between the control and macrophage repopulating colon (**Figures 8A–C**), as opposed to that seen in macrophage-depleted colons. As expected, NOLA treatment led to a decrease in resting gut diameter (main effect of NOLA treatment: F_(1,14)_ = 6.49, *p* = 0.023, n = 8 animals per group; **Figure 8B**) and colonic contraction magnitude (main effect of NOLA treatment: F_(1,14)_ = 68.6, *p* < 0.0001, n = 8 animals per group; **Figure 8C**). Interestingly, the greater increase in contraction frequency upon NOLA treatment we observed in macrophage-ablated rats at 48 hr was also noted at 7 days, despite the repopulation of Iba-1-positive macrophages (interaction effect: F_(1,13)_ = 7.72, *p* = 0.016, n = 8 animals per group, *post hoc* NOLA effect on macrophage-intact rat colon: *p* < 0.0001, *post hoc* NOLA effect on *Cx3cr1-Dtr* rat colon: *p* < 0.0001, n = 8 animals per group; **Figure 8D**). This finding suggests that Iba-1 positive macrophage repopulation was not sufficient to rescue the dysregulation of motility that occurs without inhibitory neuronal input.

## Discussion

Here we show that intestinal macrophages act to restrict intestinal motility. We also identify that this restriction of motility occurs in close interplay with neuronal inputs to the myenteric plexus. Thus, in the absence of intestinal macrophages, colonic motility was normal except when the major inhibitory neuronal input was blocked. In the absence of both this neuronal input and macrophages, colonic contractility was significantly greater than normal. Our findings suggest that this effect is maintained by CD163-positive intestinal-resident macrophages, since the restoration of the non-CD163 population failed to restore this response.

We established here that the *Cx3cr1-Dtr* rat model provides conditional ablation of intestinal macrophages upon DT injection, as for microglia and circulating monocytes in the brain as previously demonstrated (18). The repopulation of intestinal macrophages occurred by 7 days post-DT injection, in line with our previous observations for microglia in brain (18). Although we did not assess additional cell types in this study, our previous work in the ovary has shown that non-immune cell numbers are not affected (34). This rat model is therefore suitable for studying the role of intestinal macrophages in gastrointestinal function. Using this tool, we provide novel evidence that intestinal macrophages are essential in maintaining intestinal structure and that they can also regulate colonic motility in conjunction with the ENS.

This rat model has previously been characterized as a conditional microglia- and circulating monocyte- depletion model and it is striking to observe that the duration of the depletion of intestinal macrophages is very similar to that of microglia, as is the repopulation time frame (17). Cx3cr1, a microglia-associated chemokine, is highly expressed in fully differentiated and mature macrophages in the intestine (35). Notably, most intestinal macrophages in the colon appear to express Cx3cr1 (36). Bain and colleagues characterized colonic cells into those with high expression (CX3CR1^high^), those with intermediate expression (CX3CR1^int^) and those without expression of CX3CR1 (CX3CR1^-^). CX3CR1^high^ cells uniformly express F4/80 and major histocompatibility complex (MHC)II, as well as CD64, a marker that distinguishes macrophages from dendritic cells. The CX3CR1^int^ cells represent a small population of cells that are actively migrating from blood vessels as monocytes before differentiating into mature macrophages. Cells lacking Cx3cr1 expression do not express the relevant macrophage markers outlined above (36), meaning Cx3cr1 is a useful marker for intestinal macrophages and a useful target for depleting this population (17, 37–39). The similarity of microglia and intestinal macrophages extends beyond Cx3cr1 expression, as studies have identified a set of microglia-specific genes and transcription factors that are more highly similar to those of intestinal macrophages than to other macrophage subsets (35, 40). Unlike intestinal macrophages, microglia have been extensively studied for their roles in neuroinflammation and in interactions with other elements of the central nervous system (41). Therefore, the similarities of these two cell types in brain and gut may give us insights into how intestinal macrophages orchestrate gastrointestinal functions in health and disease.

Due to the heterogeneity of immune cells such as dendritic cells, T-cells, and macrophages in the intestine, it is challenging to identify and study intestinal macrophages in isolation. Thus, the ontogeny of intestinal macrophages is of great interest. There is a consensus that intestinal macrophages are continuously replenished by blood monocytes through a series of differentiation events (36, 42). However, recent studies have identified a subset of intestinal macrophages that maintain their own population, named tissue-resident macrophages (30). These self-maintaining macrophages are critical in neuroimmune interactions as they are mainly localised in the submucosal and myenteric plexuses in close proximity to enteric neurons, which in turn regulate intestinal secretion and motility (29). One of the main characteristics of these tissue resident macrophages is the expression of CD163 (30, 43). In general, Iba-1 stains for a broad subset of intestinal macrophages, including recently invaded blood monocytes as well as tissue-resident macrophages (44–46). Our data from the myenteric plexus show that most Iba-1-expressing macrophages also express CD163. This finding aligns with previous work showing that macrophages residing in the muscularis layer are predominantly tissue-resident macrophages (30, 47). A striking finding from our study was that 7 days after DT injection, most repopulating macrophages expressed Iba-1 but not CD163. There is controversy in the literature as to how macrophages are replenished in different tissues in depletion models and during natural turnover. In a lung-resident macrophage ablation *Cd169-Dtr* mouse model, tissue resident macrophages did not repopulate through CCR-2-dependent cells like monocytes, but replenish themselves locally (48). However, in another study evaluating the origin of peritoneal macrophages, Bain et al., proposed that homeostasis of resident peritoneal macrophages is achieved through a combination of self-renewal and monocyte-derived replenishment (49). In our case, we speculate that the rate of monocyte replenishment at the muscularis layer of the colon is faster than the self-renewal of tissue resident macrophages. There is also the possibility that a specific subset of tissue resident macrophages is responsible for macrophage replenishment, as it has been reported that Tim-4^+^ CD4^+^ macrophages in the intestine are capable of self-renewing (47). Further verification on whether this subtype also expresses CD163 would provide a better understanding of how intestinal macrophages maintain their population. Notably, previous studies on Kupffer cells (liver-resident macrophages) showed that once these cells are depleted in a conditional knockout model, monocytes quickly replenish and repopulate the liver but these repopulating monocytes take at least 15 days to fully express the Kupffer cells transcriptomic profile (50, 51). It is suggested that monocyte replenishment occurs in two phases, firstly via a quick replenishment phase, and secondly via a slower reprogramming/differentiation stage. We suspect similar mechanisms could also explain our observation that we did not see CD163 macrophages repopulate by 7 days after ablation. Thus, it would be interesting to undertake further immune cell analysis at later timepoints following ablation to identify if CD163-expressing macrophages repopulate and if intestinal motility function is restored as this occurs.

Macrophage sphericity often correlates with cellular activation state (52, 53). Previous studies have demonstrated that activated macrophages, which exhibit an inflammatory phenotype, have higher sphericity (52). In addition to the loss of intestinal macrophages upon DT injection, our cell analysis showed that the remaining macrophages in DT-injected rats had higher sphericity than in controls. One explanation for this observation is that DT injection predominantly ablated macrophages exhibiting lower sphericity, leaving more rounded macrophages behind. When macrophages are in a pro-inflammatory state, their Cx3cr1 expression decreases, meaning that there could be some pro-inflammatory macrophages originally present in the myenteric plexus with lower levels of Cx3cr1 (16). If this was the case, DT may be less effective at removing these pro-inflammatory macrophages leading to the observation of an increase in sphericity. Our model resulted in ablation of approximately 80% of macrophages, however, and about 40% of macrophages in the control groups had similar sphericity to those remaining after ablation. Therefore, a loss of Cx3cr1 expression and therefore a retention of pro-inflammatory macrophages is unlikely to account for the morphological cell differences we see. Another explanation for the observed higher sphericity of remaining intestinal macrophages could be that in response to the initial depletion of macrophages, the remaining macrophages may become pro-inflammatory and act to release cytokines to attract other immune cells to restore homeostasis.

Interestingly, the ablation of intestinal macrophages led to shortening of the small intestine and colon. Such gross anatomical changes are hallmarks of major intestinal disturbances such as colitis (54). However, we did not observe other features from animal models of colitis such as rectal bleeding or an increase in circulating pro-inflammatory cytokines (17). Originally, we suspected that the shortened colon length was due to a reduction in fecal pellet formation in the lumen, leaving the colons less flexible than those with more pellets. However, we did not see any difference in number of pellets inside the colon of rats with ablated macrophages compared to controls (t_(31)_ = 1.82, *p* =0.0781, n = 13 for WT and 20 for HOM/DT; graph not shown), although we did not analyze the size of the pellets, which may also influence the flexibility of the colon. It is also worth noting that macrophages have a protective role in preventing muscle atrophy as well as promoting muscle recovery, suggesting an important interaction between macrophages and skeletal muscle cells (55). The protective role of macrophages could also explain our observation that shortening of the small intestine and colon was rescued upon repopulation of intestinal macrophages. Further histological examination of structures in the gastrointestinal tract such as the mucus lining and muscle thickness in the absence of intestinal macrophages will be important considerations in the future.

Our findings reveal that intestinal macrophages are not crucial for colonic motility under control conditions whereby contraction frequency was not affected by the absence of these cells. While several studies have demonstrated that intestinal macrophages influence gastrointestinal dysmotility in disorders such as inflammatory bowel disease and post-operative ileus (56–58), to our knowledge only two studies have demonstrated an impact on colonic motility under control (homeostatic) conditions (6, 30). In the study by Muller et al., *ex vivo* colonic motility was measured as contraction force generated by a 3 mm colonic ring, followed by a stretch stimulus in adult mice (6). De Schepper et al. measured gastrointestinal motility via ileal muscle strip contractility, gastrointestinal transit and gastric emptying (30). In addition to differences in mechanical measurements, these studies investigated the effects of depleting macrophages at the embryonic phase, not acutely in adulthood as in our work (6, 30).

Another important finding from the present study is that intestinal macrophages are crucial in regulating colonic motility when NOS is inhibited. Blockade of nNOS depletes the major inhibitory signal in the ENS so that smooth muscles are excited at a higher frequency (59). Under these circumstances, intestinal macrophages may act to regulate and even prevent hyper-contraction of the colon. In our study, we observed that macrophage-ablated colons had a much higher increase in contraction frequency upon NOLA treatment when compared to controls. This indicates that intestinal macrophages have an additional inhibitory role in modulating gastrointestinal physiology and reveals their importance specifically in regulating colonic motility. Since the CD163-positive resident intestinal macrophages did not repopulate at the 7-day post DT injection timepoint, we suspect that this subtype of resident intestinal macrophage is crucial in inhibiting colonic motility in addition to the inhibitory neuronal input from the ENS, supporting previous reports that self-maintaining resident macrophages are essential for gastrointestinal transit (30). Although we did not observe changes in neuronal numbers or proportions of nNOS neurons within the myenteric plexus, macrophage depletion could lead to apoptosis of neurons that would not be reflected in Hu/NOS immunostaining alone (60). Notably, our findings suggest that intestinal macrophages can influence contraction frequency but do not affect contraction magnitude. In general, both parameters involve neural-muscular transmission from the myenteric plexus to smooth muscle, under the control of interstitial cells of Cajal (61). The contraction magnitude is chiefly the outcome of the excitation and relaxation of longitudinal muscle and circular muscle, while contraction frequency is mainly determined by the neural input in response to physical tension (14). Therefore, our results imply that intestinal macrophages can directly interact with enteric neurons to exert an inhibitory effect on contraction frequency even when inhibitory neural input is significantly reduced. In terms of a mechanism for this, it has previously been reported that intestinal macrophages can interact with smooth muscle layers via the transient receptor potential cation channel subfamily V member 4 (TRPV4)- prostaglandin E2 (PGE-2) axis, the IL-17A-iNOS axis or CSF-1/BMP-2 crosstalk with neurons (3). Purinergic neurotransmission may also play a role in the inhibitory regulation of gut motility (62, 63). P2X receptors are expressed in the submucosal plexus, myenteric plexus, as well as the smooth muscle layers (64). In particular, P2X2R receptors localized in intermuscular neurons are involved in the regulation of smooth muscle contraction (65). Therefore, it would be of interest to assess how CD163-expressing macrophages regulate colonic motility through potential downstream effects on these pathways.

In conclusion, this is the first study examining the role of intestinal macrophages in a conditional macrophage ablation rat model and the first such study to utilize *ex vivo* video imaging techniques to assess colonic motility in these rats. Our findings highlight the importance of intestinal macrophages in maintaining gastrointestinal structure and illustrate that tissue resident macrophages are likely to regulate colonic motility in the absence of inhibitory neuronal input. Gastrointestinal disorders where inhibitory neuronal input is suppressed, such as gastroparesis and achalasia, are often caused by bacterial or viral infection with involvement of macrophages (66, 67). Our evidence implicating a role for intestinal macrophages gives insight into how pathophysiology may manifest in these conditions. Future directions should focus on dissecting the precise mechanism of how intestinal macrophages regulate colonic motility and differentiating the subtypes of intestinal macrophages involved in supporting normal intestinal structure. A better understanding of the role of intestinal macrophages will provide macrophage-specific therapeutic targets for various gastrointestinal disorders.

## Data availability statement

The raw data supporting the conclusions of this article will be made available by the authors, without undue reservation.

## Ethics statement

The animal study was approved by RMIT University Animal Ethics Committee (AEC #1920). The study was conducted in accordance with the local legislation and institutional requirements.

## Author contributions

JY: Data curation, Formal analysis, Investigation, Methodology, Validation, Visualization, Writing - original draft. GB: Methodology, Supervision. SX: Investigation. EH-Y: Conceptualization, Funding acquisition, Supervision, Resources, Writing - review & editing. SS: Conceptualization, Funding acquisition, Supervision, Resources, Writing - review & editing.
